# Challenging diagnosis of congenital hyperinsulinism in two infants of diabetic mothers with rare pathogenic *KCNJ11* and *HNF4A* gene variants

**DOI:** 10.1186/s13633-018-0060-7

**Published:** 2018-07-17

**Authors:** Lina Huerta-Saenz, Carol Saunders, Yun Yan

**Affiliations:** 1 Children’s Mercy Kansas City, Division of Pediatric Endocrinology, 3101 Broadway Blvd, Kansas City, MO 64111 USA; 2 Children’s Mercy– Wichita Specialty Clinic, Wichita, KS USA; 3 University of Missouri-Kansas City, Kansas City, MO USA; 4 University of Kansas Medical Center-Wichita School of Medicine, Wichita, KS USA; 5 Center for Pediatric Genomic Medicine Children’s Mercy Hospital, Kansas City, MO USA; 6 Department of Pathology and Laboratory Medicine, Children’s Mercy Hospital, Kansas City, MO USA; 7 Present address: Penn State College of Medicine, Penn State Children’s Hospital- Division of Pediatric Endocrinology and Diabetes, Hershey, PA USA

**Keywords:** Congenital Hyperinsulinism, Infant of diabetic mother, Neonatal hypoglycemia

## Abstract

**Background:**

Congenital hyperinsulinism (CHI) is the leading cause of persistent hypoglycemia in infants. The infants of diabetic mothers (IDMs) very frequently present with neonatal hypoglycemia associated to transient hyperinsulinism however the incidence of CHI in IDMs is unknown.

**Case presentation:**

Here we report 2 cases of CHI where the diagnoses were challenged and delayed because both patients were infants of diabetic mothers (IDMs) and had concomitant complicated medical conditions. Case 1 was heterozygous for a likely pathogenic variant in *KCNJ11*(p.Arg206Cys), and Case 2 was heterozygous for a pathogenic *HNF4A* variant, (p.Arg267Cys). *HNF4A*-associated CHI is very rare, and this particular case had a clinical phenotype quite different from that of previously described *HNF4A*-CHI cases.

**Conclusions:**

This case series is one of few reports in the medical literature describing two IDMs with persistent recurrent hypoglycemia secondary to CHI, and a different clinical phenotype for *HNF4A*-associated CHI. IDMs typically present with transient hyperinsulinism lasting no more than 2–3 days. Since being an IDM does not exclude CHI, this diagnosis should always be considered as the mostly likely etiology if neonatal hypoglycemia persists longer than the described time frame and genetic testing for CHI confirmation is highly suggested.

## Background

Congenital hyperinsulinism (CHI) is the leading cause of persistent hypoglycemia in infants, causing neurodevelopmental and cognitive delays in up to 25–50% of affected children [1]. Early recognition and appropriate treatment are important to prevent such adverse outcomes.

The estimated incidence of CHI is reported to be as low as 1/50,000 live births in Holland to as high as 1/2500 in geographic areas where consanguinity is highly frequent, such as Saudi Arabia [2]*.* CHI is caused by genetic defects that cause disease through changes in channels expression/activity, transcription factors expression and disturbed enzyme activities. More frequently is increased activity rather than enzymes deficiency All these defects cause the common finding of recurrent episodes of hyperinsulinemic hypoglycemia due to an inappropriate secretion of insulin by the pancreatic beta-cells [3].

In 2016, there were 11 genes known to be associated with monogenic forms of CHI (*ABCC8*, *KCNJ11, GLUD1, GCK, HADH1, UCP2, MCT1, HNF4A, HNF1A, HK1*, and *PGM1*) [4, 5]. Variants in the *ABCC8* and *KCNJ11* genes together comprise approximately 90% of the identified cases of diazoxide-unresponsive CHI [6]. These genes encode SUR-1 and Kir6.2, respectively, which comprise two subunits of K_ATP_-HI, the beta-cell plasma dependent membrane ATP-potassium channel [7, 8]. Most pathogenic variants in these 2 genes are recessive and cause loss of function; however, some cases of dominantly inherited inactivating mutations have been reported. In the past, clinical phenotype of patients with dominant variants was different from those with recessive variants but the biochemical phenotype was not different [9]. However, in 2013 Snider et al. published a large cohort of 417 children with CHI whose genotype-phenotype correlations were most successful if they have GLUD1, GCK, and recessive K_ATP_ mutations [6]. Many of these correlations were challenging due to the high frequency of novel missense K_ATP_ mutations because these defects might be either recessive or dominant and if dominant these could be either responsive or unresponsive to diazoxide. In these cases, mutation analysis including parental testing should be considered. However, the existence of novel K_ATP_missense mutations variability (recessive/dominant) in adult carriers is typically unknown because they are frequently asymptomatic unless challenged with fasting or protein tolerance test [6, 9, 10].

Diazoxide, the first-line treatment for CHI, acts by opening the K_ATP_-channel and decreasing insulin secretion [11]. The effect of the gene variant on K_ATP_-channel expression determines the clinical phenotype and the response to diazoxide [4]. Octreotide, a second-line agent, is a long-acting somatostatin analog that decreases insulin secretion through hyperpolarization of the beta-cells and inhibition of calcium channels but has been associated with treatment failure due to tachyphylaxis and fatal necrotizing enterocolitis [8].

There are two distinct histologic forms of CHI: focal and diffuse. In a published cohort of 52 infants with CHI treated from 1985 to 1998, twenty-two had focal CHI and the rest had diffuse CHI. Only one neonate with focal HI and two with diffuse HI responded to treatment with diazoxide. All the other infants require pancreatic surgery to achieve cure [12].

Variants in a third gene, *HNF4A,* are associated with transient CHI in early infancy, despite also causing maturity-onset diabetes of the young (MODY 1) [13]. *HNF4A* variants are typically rare and the incidence varies per population but have been described as the third most common cause of diazoxide-responsive CHI in a large cohort of 300 patients with CHI from United Kingdom [14, 15].

The infants of diabetic mothers (IDM) born with macrosomia typically present frequent metabolic complications including hypoglycemia, jaundice, respiratory distress, asphyxia and are in greater need of intensive care as reported by Looreda-Garcia et al. in a large cohort of 18,005 newborns where 10% were infants of diabetic mothers [16]. The hyperinsulinemic hypoglycemia seen in IDMs occurs secondary to transient hyperinsulinism but it does not last longer than 2 or 3 days [7].

The current 2011 American Academy of Pediatrics guidelines for postnatal glucose homeostasis in healthy late-preterm and term infants mandate that persistent hypoglycemia, lower than 25 mg/dL from birth to 4 h of age, or lower than 35 mg/dL from 4 to 24 h of age after attempts to reefed, requires treatment with intravenous (IV) glucose [17]. These prior AAP guidelines focused on when to screen infants for hypoglycemia and when to provide treatment but did not address the etiology work up procedure for prolonged and recurrent neonatal hypoglycemia. The Pediatric Endocrine Society recommendations published in 2015 considered the interpretation and response to plasma glucose (PG) concentration during the first 2 days of life is controversial however the mean PG concentration in normal newborns increases with time and by 72 h of age is similar to those in older infants and children. Therefore, these guidelines suggest delaying diagnostic evaluations until 2–3 days after birth [17].

Here we report 2 cases of IDMs with rare genetic variants of CHI whose final diagnoses of CHI were complicated and delayed because the patients were IDMs and had concomitant challenging medical conditions.

## Cases presentation

### Case 1

A Hispanic male infant was born large for gestational age (LGA) (birth weight 4.11 kg, above 100th percentile and length 51 cm, 98.6th percentile) at 34 weeks to a 17-year-old mother with uncontrolled gestational diabetes and pre-eclampsia. No family history iof hypoglycemia was reported. He was born by emergency C-section at an outside medical center when fetal monitoring revealed a flat strip. At the time of delivery, his APGAR scores were 2 at 1 min, and 6 at 5 min. His initial arterial blood gas was pH 7.13, pO2 65, pCO2 40 mmHg, base excess (− 9.2). His initial plasma glucose level was 7 mg/dL. He received an IV bolus with D12.5%, followed by IV fluids (IVF) with a glucose infusion rate (GIR) of 6.9 mg/kg/min. The infant was transferred to our hospital at day of life (DOL) 2 for respiratory distress syndrome (RDS), supraventricular tachycardia, and hypoplastic aortic arch as well as persistent hypoglycemia.

During his hospital stay, the GIR was gradually increased to 16 mg/kg/min. Polycose supplement was added to his feeds with the goal of maintaining plasma glucose levels close to 70 mg/dL when titrating IV dextrose. However, on DOL 21, he developed hypoglycemic episodes (BG below 50 mg/dL) after IV dextrose was weaned off and despite consuming 26 cal/oz. of formula. Extensive workup for CHI was initiated on DOL 25, and the results revealed an elevated insulin level of 16.3 mcIU/mL coincident with a plasma glucose level of 48 mg/dL and beta hydroxybutyrate level of less than 100 mcmol/L. Ammonia, pyruvic acid, cortisol level,, serum amino acids, acyl carnitine profile, and urine organic acids were all in the normal range (Table 1). A pediatric endocrinology consultation was performed and DNA sequencing for CHI was ordered, revealing a heterozygous maternally-inherited likely pathogenic variant in *KCNJ11,* c.616C > T (p.Arg206Cys). This variant has been previously reported in a family where it segregated with several affected individuals with CHI. It is rare in population databases (ExAC and gnomAD; 3/242,646 alleles) [18] and predicted by in silico tools (GERP, SIFT, PolyPhen) to be damaging [19]. Diazoxide was started and plasma glucose levels stabilized above 70 mg/dL at the dose of 15 mg/kg/day on DOL 30. The patient was discharged home at DOL 71 with diazoxide at 10 mg/kg/day. His plasma glucose was stable on a similar dose at the time of his last visit when he was 11 months old.

**Table 1:** Critical Sample Laboratory Data of Case 1 (*KCNJ11* gene mutation)

Parameter (DOL #26: 2/26/2013)	Laboratory Value	Normal Range
POC Blood glucose	48 mg/dL	70–99 mg/dL
Serum Blood Glucose	58 mg/dL	70–99 mg/dL
Serum Cortisol	23 mcg/dL	7–25 mcg/dL
Serum Growth Hormone	4.6 ng/mL	0–7 ng/mL
Serum Insulin	16.3 mcIU/mL	2–18 mcIU/mL
Beta Hydroxybutyrate	Less than 100	0–269 mcmol/L
Urinary Ketones	N/A	N/A
Serum Lactic Acid	1 mmol/L	0.7–2.1 mmol/L
Serum Pyruvic Acid	0.08 mmol/L	0.08–0.16 mmol/L
Free Fatty Acids	Not collected	N/A
Plasma Acyl carnitine profile^a^	Normal	
Urine Organic Acids^a^	Normal	
Serum Ammonia^a^	10 mcmol/L	10–62 mcmol/L
Serum Amino Acid Profile^a^	Normal	N/A

(^a^) Samples were collected on DOL#46 (03/05/2013)

### Case 2

A male infant was born small for gestational age (SGA) [birth weight 1.45 kg (4.7th percentile), and birth length, 40 cm (3.5th percentile)] at 33 weeks of gestational age to a 35-year-old mother with gestational diabetes and pregnancy-induced hypertension (PIH) at a local medical center. His mother managed her gestational diabetes with diet control only. Family history was remarkable for type 2 diabetes in the maternal grandmother. No family history of hypoglycemia reported. Paternal history was unknown. His APGAR scores were 1, 6, and 10 at 1, 5, and 10 min, respectively. He developed persistent hypoglycemia on DOL 5 (Plasma Glucose = 43 mg/dL) and also had transient RDS.

Treatment for hypoglycemia was started with dextrose 10% IVF with GIR at 6–10 mg/kg/day in addition to increased oral feeds. He also received hydrocortisone treatment (30 mg/m^2^/day) secondary to low cortisol level during a prior hypoglycemic event (4.3 mcg/dL) from DOL 12 to DOL 20. Hypoglycemia recurred after discontinuing hydrocortisone therapy as well as dextrose containing IVF, and then our pediatric endocrinology team was consulted. Critical labs were obtained on DOL 21 (Table 2). Urine ketones were negative but the beta hydroxybutyrate was not obtained at outside hospital. The physical exam of this infant was negative for concerning findings for hypopituitarism.

**Table 2:** Critical Sample Laboratory Data of Case 2 (*HNF4A* gene mutation)

Parameter	Laboratory Value	Normal Range
POC Blood glucose	43 mg/dL	70–99 mg/dL
Serum Blood Glucose	46 mg/dL	70–99 mg/dL
Serum Cortisol	8.3 mcg/dL	7–25 mcg/dL
Serum Growth Hormone	11.3 ng/mL	0–7 ng/mL
Serum Insulin	1.5 mcIU/mL	3–25 mcIU/mL
Beta Hydroxybutyrate	N/A	N/A
Lactic Acid	1.6 mmol/L	0.5–2.2 mmol/L
Ammonia	38 mcmol/L	11–32 mcmol/L

High-dose ACTH stimulation testing (abbreviated) was performed due to random low cortisol level and the infant responded adequately with a stimulated cortisol level at 29.7 mcg/dL and baseline cortisol level at 10.4 mcg/dL. Serum ammonia and lactic acid levels were normal. A glucagon challenge test was recommended by the pediatric endocrine team but was not performed. The infant was started on diazoxide at 15 mg/kg/day with good response. DNA sequencing for CHI revealed a heterozygous pathogenic missense variant in exon 7 of *HNF4A*,( p.Arg267Cys; c.799C > T; legacy Arg245Cys). This variant affects a highly conserved amino acid and is predicted to be deleterious by in silico tools. It has been previously detected in patients with MODY 1 [20], and different substitutions affecting this same codon (p.Arg267Ser and p.Arg267Gly) have been reported [21]. This variant is absent from population databases including GnomAD, ExAC and Leiden Open Variation Database. Parental testing was not performed due to maternal refusal and unavailability of father. Renal ultrasound, liver function tests, and urinalysis of this patient were normal. The patient responded well to diazoxide, which was gradually tapered based on POC home glucose values in the range of 70–100 mg/dL and hemoglobin A1C, and eventually discontinued at 6 months of age. Fasting challenge test was not performed before total discontinuation of diazoxide. Infant remained euglycemic at the time of his last visit in clinic (2 years 9 months of age).

## Discussion

In these 2 cases, the diagnostic workup for CHI was complicated by their status as infants of diabetic mothers. For both, the standard treatment for neonatal hypoglycemia was provided but the specific work up for CHI was not performed until both infants were older than 3 weeks of life. Case 1 had perinatal asphyxia, prematurity, and sepsis as his underlying diagnoses and therefore his hypoglycemia was initially considered to be secondary to these underlying diagnoses rather than due to CHI, and Case 2 was presumed to have transient hyperinsulinism due to his small for gestational age condition, in addition to being an IDM with concomitant PHI.

The incidence of CHI in IDMs is not well known considering the absence of published reports in the literature. However, considering IDMs have higher rates of prolonged hospitalization at the NICU services is remarkably important to bring these findings to the attention of pediatricians and neonatologists to increase awareness about the need of considering CHI as the main etiology of prolonged and recurrent hypoglycemia in all neonates including the infants of diabetic mothers.

The most common risks for neonates developing abnormal postnatal glucose homeostasis include being SGA, LGA, IDMs, history of perinatal asphyxia and prematurity [17]. Case 1 was born LGA, IDM, premature and had asphyxia at birth, leading perinatal stress-induced hyperinsulinism (HI) to be included on his differential diagnosis. Stress-induced HI can be seen in asphyxia, as well as maternal toxemia and intrauterine growth retardation [22]. This former medical condition can persist for several weeks after birth, but it usually remits spontaneously. Perinatal asphyxia in Case 1 was probably one of the confounding factors contributing to the delay in considering CHI as the most likely etiology of his prolonged and recurrent hypoglycemia. Case 2 was also an IDM, premature but born SGA and these three risk factors played an important role in the delay of considering CHI as the final etiology of his persistent neonatal hypoglycemia.

Pertinent workup for the etiology of persistent neonatal hypoglycemia includes obtaining critical samples in a timely manner and performing genetic testing. Critical samples for evaluation of hypoglycemia include plasma glucose, beta-hydroxybutyrate, carbon dioxide, lactate, free fatty acids, cortisol, growth hormone, total/free carnitine, acyl carnitine profile, C-peptide, and pro-insulin levels. In 2015, the Pediatric Endocrine Society (PES) of the United States published new recommendations for evaluation and management of persistent hypoglycemia in neonates, infants, and children and the specific timeframes to start performing work up for CHI were also described [23]. Adherence to the current PES recommendations in all NICUs and nurseries would potentially avoid delays on neonatal hypoglycemia etiology work up and this potentially would lead to a prompt diagnosis of CHI. Prior AAP guidelines published in 2011 failed to address the diagnosis and management of disorders causing recurrent and prolonged hypoglycemia beyond the neonatal period [17].

DNA sequencing is remarkably important when CHI is suspected because it contributes to accurate medical treatment, genetic counseling, potential distinction between focal and diffuse disease, determination of the likelihood of good response to medications, and consideration of early pancreatic surgery. In a cohort study from the United Kingdom of infants who underwent rapid sequencing, 27 patients (26%) were positive for *ABCC8* variants and 5 of them (5%) were positive for *KCNJ11* variants [24]. Of the 5 with *KCNJ11*-variants, 2 underwent subtotal pancreatectomy. A significant proportion (18%) who tested positive for *ABCC8* or *KCNJ11* variants achieved euglycemia with medical treatment. All infants with maternal heterozygous mutations achieved remission, leading to the conclusion that maternally inherited variants may confer a higher rate of remission.

Genetic testing is also very helpful in CHI because even though patients with inactivating mutations of GCK (MODY 2), inactivating mutations of hepatocyte nuclear factor*HNF1A *(MODY 3) and *HNF4A* (MODY 1) can present as congenital HI in neonates they can later evolve in early adulthood into insulinopenic diabetes; and activating mutations of the KATP channel subunits can cause either severe permanent neonatal diabetes or a mild increased susceptibility to adult-onset diabetes [5].Case 1 was heterozygous for a maternally inherited likely pathogenic variant in *KCNJ11*. This variant has been reported in one family where it segregated with disease in four symptomatic hypoglycemic individuals [19]. It is rare (present in only 2 of ~ 12,000 alleles) in the general population and predicted by in silico tools to be damaging. However, this is the first time this variant has been reported in an IDM.

The phenotype of Case 2 (SGA) did not follow the classical phenotype of IDMs who are typically born with macrosomia. Most infants reported with *HNF4A* variants had increased birth weight and macrosomia [25], and they also had transient CHI requiring diazoxide therapy between 3 months and 8 years [26]. Case 2 was born SGA in contrast to the prior reports of *HNF4A*-associated CHI infants (E.g. p.R63W mutation) who are typically born with macrosomia [26]. However, one recent report of an infant with *HNF4A*-CHI and carrier of the p.R63W mutation described that infant was born with normal weight but this infant was not an IDM [27]. It is not clear if the SGA phenotype of Case 2 was secondary to his underlying *HNF4A* variant, his prematurity (considering that insulin is an important growth factor in the third trimester of pregnancy) or if this was a consequence of the intrauterine growth retardation associated with his maternal PIH.

With regard to his response to medical treatment, diazoxide therapy in Case 2 was rapidly tapered and successfully discontinued similar to the prior *HNF4A* case reported by Flanagan in 2010 [14].

It is important to describe this transient hyperinsulinism clinical course on Case 2 because his successful response to diazoxide could be associated to his R267C gene variant, and could be also linked to future progression to MODY. Even though we recommended genetic testing for both parents, and mother was referred to genetics for testing and counseling (father was not available and his family medical history was unknown) she was not able to pursue genetic testing due to unavailable medical insurance coverage. We discussed with mother the potential implications of genetic testing for her diagnosis (she has high risk to be a HNF4A carrier) but she reported to be healthy and refused further evaluation with OBGYN.

## Conclusions

This case series is one of the few reports describing two IDMs with persistent recurrent hypoglycemia secondary to genetic CHI, and an atypical clinical phenotype for *HNF4A*-associated CHI. Prompt consideration of CHI as the most likely etiology of recurrent and prolonged neonatal hypoglycemia even in IDMs is important to facilitate prompt consultation with endocrinology, and appropriate timely workup including genetic testing as recommended by the current PES clinical guidelines [23]. Transient neonatal hypoglycemia in an IDM is not expected to last more than 2–3 days. In all the cases of neonatal hypoglycemia persisting beyond this timeframe, including infants of diabetic mothers, CHI should be considered as the presumed etiology.

## Abbreviations

CHI: Congenital hyperinsulinism
DOL: Day of life
ES: Exome sequencing
GIR: Glucose infusion rate
IDM: Infant of a diabetic mother
IVF: Intravenous fluids
LGA: Large for gestational age
PG: Plasma glucose
PIH: Pregnancy induced hypertension
RDS: Respiratory distress syndrome
SGA: Small for gestational age
